# Successful Intravascular Lithotripsy Within a Covered Coronary Stent

**DOI:** 10.1016/j.jscai.2025.103733

**Published:** 2025-06-24

**Authors:** Michael S. Cross, Vinh Duong, Shiv K. Agarwal, Barry F. Uretsky

**Affiliations:** a Cardiology Section, Central Arkansas Veterans Healthcare System, Little Rock, Arkansas; b Cardiology Division, University of Arkansas for Medical Sciences, Little Rock, Arkansas; c Stern Cardiovascular Foundation, Memphis, Tennessee; d Atlanta Heart Associates, Atlanta, Georgia

**Keywords:** coronary perforation, covered stent, intravascular lithotripsy, percutaneous coronary intervention, rotational atherectomy

Intravascular lithotripsy (IVL) has become an important method to treat calcified lesions.^1^ More recently, IVL has been demonstrated to allow for increased luminal gain in previously placed coronary stents.^2^, ^3^, ^4^ IVL has not been systematically studied within covered stents either in vitro or in vivo. In this image series, IVL was used to expand an underexpanded covered stent implanted after coronary perforation during rotational atherectomy. We hypothesized that the covered stent material did not significantly attenuate the energy impulse from IVL, allowing adequate energy to be transmitted to allow for calcium fracture. We present images demonstrating progressive stent expansion with each series of pulses, which allowed effective postdilation of the covered stent.

**Figure 1:**
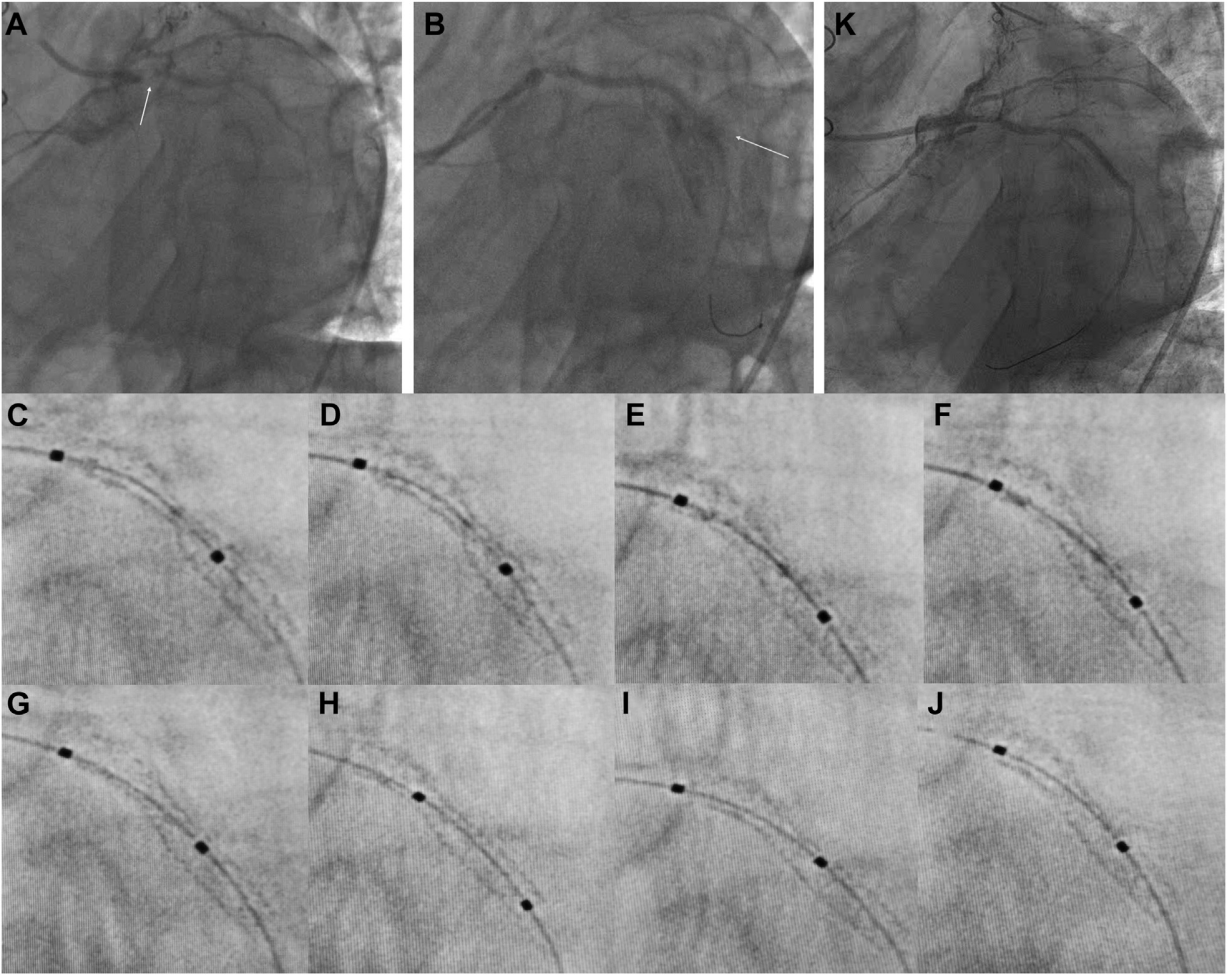
**Image of underexpanded covered stent placed after peration from rotational atherectomy and subsequent covered stent expansion with intravascular lithotripsy (IVL).** (A) Prepercutaneous coronary intervention image showed severe and diffuse disease of the circumflex. There was a 95% proximal circumflex lesion (arrow). (B) With rotational atherectomy, an Ellis III perforation developed (arrow shows extravasated contrast). (C-J) The progression of covered stent expansion is demonstrated after each of the 8 Shockwave treatments (10 pulses in each) from panels C to J, as captured by StentBoost (Philips). Please note that after final pulses, stent appears essentially fully expanded. (K) Postintravascular lithotripsy angiogram shows fully expanded stent.

A 75-year-old man was transferred from an outside hospital with an non−ST-elevation myocardial infarction. Angiography revealed severely calcified 90% ostial and proximal left circumflex lesions with poor distal flow (Figure 1A, Supplemental Video 1). The proximal lesion was not dilatable with high-pressure, noncompliant balloons. Rotational atherectomy with a 1.25-mm burr (Boston Scientific) resulted in an Ellis type III perforation (Figure 1B, Supplemental Video 2). Balloon tamponade was ineffective in sealing the perforation. A 2.5-mm × 20.0-mm Papyrus covered stent (Biotronik) was deployed and contained the perforation, but the stent was underexpanded. Despite 2 postdilation attempts with a noncompliant balloon at high-pressure (>16 atm), the covered stent remained underexpanded with TIMI grade 1 flow distally.

The patient was brought back 2.5 months later for IVL of the underexpanded covered stent. A 2.5-mm × 12.0-mm IVL balloon (Shockwave Medical) was passed easily and inflated within the covered stent where 80 pulses were delivered with progressive stent expansion (Figure 1C-J). A 2.75-mm × 12.0-mm noncompliant balloon was then used for postdilation. Postinterventional angiography showed excellent results (Figure 1K, Supplemental Video 3).

IVL’s role continues to expand as a novel and effective method for treating in-stent restenosis and stent underexpansion due to severe calcification.^2^, ^3^, ^4^ This case demonstrates the successful utilization of IVL as sonic pressure waves could still pass through the polyurethane covering material of the underexpanded covered stent and fracture luminal calcification, allowing for stent expansion.^5^
